# Inside the Bell Jar of Social Media: A Descriptive Study Assessing YouTube Coverage of Psychotropic Medication Adherence

**DOI:** 10.3390/ijerph20166578

**Published:** 2023-08-15

**Authors:** Aysha Jawed, Nadia Zaim

**Affiliations:** 1 Johns Hopkins Children’s Center, Baltimore, MD 21287, USA; 2 Department of Pediatric Social Work, Johns Hopkins Children’s Center, Baltimore, MD 21287, USA; 3 Johns Hopkins University School of Medicine, Baltimore, MD 21205, USA; nzaim1@jhmi.edu; 4 Department of Psychiatry and Behavioral Sciences, Division of Child and Adolescent Psychiatry, Johns Hopkins University School of Medicine, Baltimore, MD 21205, USA

**Keywords:** psychotropic medication, treatment adherence, side effects, psychiatric disorder, stigma, digital health

## Abstract

The global mental health crisis is a longstanding one that impacts a multitude of patient populations worldwide. Within this crisis, psychiatric medication adherence is yet another complex public health challenge that continues to persist and contribute towards the chronic nature of the increased incidence and prevalence of psychiatric morbidities, which in turn result in the sequalae of substantial costs to humanity, the healthcare system, lost productivity, functioning and disability among patients with mental disorders. Psychotropic medication adherence is a significant part of psychiatric care and treatment across severity levels of mental illness. This health behavior is also filled with complexities, given the abundance of social and behavioral determinants as well as intrinsic and extrinsic factors that surround this health behavior. Examining contexts for promoting this health behavior change is crucial in determining directions for addressing it more optimally. There have been several published studies on considerations and interventions to address this health behavior; however, to date, no studies have been published on assessing coverage and directions of content across social media platforms, which trend as a rising health communication medium in our digital era. The present study is the first of its kind to dive into exploring the nature of widely viewed content and deliverers of this content on a prominent social media platform, YouTube, as the basis to determine potential directions for future intervention that can extend to reaching more patients struggling with this high-risk health behavior across the world, given the global reach of social media.

## 1. Introduction

Nearly 970 million individuals are affected by psychiatric illness worldwide [1]. Psychiatric illness has been consistently associated with decompensation in functioning, suboptimal quality of life, lost productivity, exacerbation of illness and substantial costs to humanity, the healthcare system, and society [2,3]. Psychiatric illness has exacerbated within the growing mental health crisis across the world, especially during the past couple of years with the emergence of the global COVID-19 pandemic and the sequalae that followed thereafter [2].

Psychotropic medication adherence is a complex health behavior affected by a range of social and behavioral determinants as well as intrinsic and extrinsic factors [3]. There are multiple classifications of psychotropic medications [4,5]. The most prevalent ones include antidepressants, antipsychotics, anti-anxiety medications, mood stabilizers, and stimulant medications [5]. Many psychotropic medications can be utilized for treatment of greater than one psychiatric morbidity [6,7,8]. There continues be significant stigma surrounding the utilization of these medications [9,10,11]. Further, it is an established fact that patients with chronic, progressive medical illness are more susceptible to developing psychiatric morbidities [12,13,14,15]. In addition, it is well known that untreated psychiatric illness can impact academic, social, and developmental domains of life [16,17]. Nevertheless, these grappling realities have not sufficiently resulted in critical examination in addressing this trending public health crisis that has exacerbated within our current global mental health crisis.

Several published studies have explored different intrinsic and extrinsic factors affecting psychotropic medication adherence as targets for intervention in different in-person and telehealth contexts [18,19,20,21,22,23,24]. However, to date, no studies have examined coverage of psychotropic medication adherence on social media spaces, which continue to rise as a health communication medium in our contemporary digital health landscape. Based on a recent study by the Pew Research Center, the most prevalent social media platforms trending at this time for acquisition of content surrounding everyday life considerations are YouTube, Facebook, and Instagram [25]. Notably, YouTube has attracted the most viewers across demographic groups, given its increased accessibility of content stemming from the public nature of it [25].

Several clinical modalities have been utilized over time to assess for medication adherence across both medical and psychiatric illnesses. Cognitive behavioral therapy and motivational interviewing are among them [26]. In the context of our digital era, both of these forms of therapeutic interventions are also accessible on a telehealth basis [21]. Given the digital landscape, nontraditional sources of support via technology to address different determinants surrounding this complex health behavior could be helpful in determining degree of viewer engagement as a predictor of potential targets for intervention in heightening treatment adherence. It follows that social media, as another nontraditional communication medium in healthcare, yields promise in achieving a more developed understanding of determinants to inform further intervention for this longstanding public health crisis. Furthermore, alongside academic and governmental literature, social media could also be an education medium in in our digital health era to uncover a diversity of perspectives and management options for varying severity levels of mental illness.

Our study was the first of its kind to critically examine the diversity of content on this health behavior across a prominent social media platform, but not to establish causality or assess the efficacy of interventions implemented on social media; rather, this study sought to uncover the existing stage of coverage related to a significant part of psychiatric care and treatment on a globally accessible social media platform. The goals of this study were the following: (1) present the sources and formats of the widely viewed videos on psychotropic medication adherence across YouTube; (2) critically assess the content across these widely viewed videos; (3) and provide recommendations for further research and practice endeavors in focusing on this health behavior as a target for intervention across social media platforms in our trending digital health landscape.

## 2. Materials and Methods

The research design of the present study was cross-sectional in nature and involved collecting observational data at one conceptual point in time from the YouTube social media platform. From April to May 2023, browser history on computers of the two authors was cleared. Next, the researchers conducted a search on YouTube using permutations of specific key words and descriptors embodying psychiatric or psychotropic medication adherence or compliance. Piloting various key words (taking/take psych medication, psychotropic, psychiatric, adherence, compliance) was instrumental in ultimately narrowing the range of phrase(s) that yielded the most relevant videos, highest view counts across videos, and greatest cumulative views for the top 30, 60, and 100 videos, respectively. After completing these pilot searches, the key words that formed the search strategy were “take psych medication”, which yielded the most widely viewed videos that were directly relevant to psychotropic medication adherence. Notably, psychotropic or psychiatric medication adherence (or compliance) yielded significantly fewer videos with low view counts. The results were subsequently filtered by view count, and the URLs for the 100 most widely viewed videos were safeguarded in a separate file. Overlapping URLs were deleted and replaced. In turn, only one URL for each video was kept for coding and analysis.

The researchers then created a codebook based on a review of literature and guidelines from authoritative and expert sources such as the American Psychiatric Association, American Psychological Association, and the Diagnostic and Statistical Manual of Mental Disorders. Each researcher viewed and coded all videos between April and May in 2023. Intra- and inter-rater reliability of the coding was demonstrated for each researcher and between them, respectively. The following information was reviewed and coded for each video: (a) source of upload, (b) format, (c) number of views, (d) length (in minutes), (e) year of upload, and (f) content. IRB approval was not required for this study given that the videos are publicly available.

### 2.1. Eligibility Criteria

All videos reviewed were in English. Any videos not narrated or presenting written content in English were excluded from analysis. Content across all videos pertained to psychotropic medication adherence. The researcher viewed the full video, which constituted the unit of analysis. There were no parameters for consideration of videos based on length of time.

### 2.2. Measurements and Coding Specifications

The instrument included the following basic information: coder, video identification number (which was assigned), date the video was uploaded, date the video was coded, length of video (in minutes), number of views, and title of the video. Next, there were three sections for source of upload, format and content in the instrument. Content included several content categories comprising many variables (discussed below), all of which were coded dichotomously (i.e., either yes or no) to indicate presence or absence across each video.

The source of upload for each video was coded into one of the following four categories: organizational, consumer, governmental, and other sources. The categories for coding format included Documentary; Interview; Demonstration/Experiment; Talk by Professional; TV Talk Show/Discussion panel; Animation; Still images; News report with anchor; V-blog; Advertisement; Testimonial/Story; Multiple formats; and Other formats. The following 15 content categories were created in this codebook: (a) type of medication; (b) psychiatric diagnoses; (c) stressors; (d) health beliefs; (e) special considerations; (f) community resources; (g) support networks; (h) side effects of medication use; (i) stigma; (j) personal factors; (k) developmental/age groups; (l) additional interventions; (m) access to medications; (n) additional factors; (o) medication adherence assessment tools; and (p) open-ended comments on misinformation or disinformation depicted in the video. Conceptualization of the codebook involved developing these content categories to account for the comprehensive range of targets for intervention from addressing psychotropic medication adherence.

### 2.3. Demonstration of Intra- and Inter-Rater Reliability

The researchers demonstrated both inter- and intra-rater reliability of the data from coding content across all videos in the sample. To demonstrate intra-rater reliability, the researcher utilized a random number generator to randomly selected 10 videos and recoded them within 2 weeks of the original coding. This analysis included all of the dichotomously coded (Yes versus No) content variables in the instrument. Intra-rater reliability was found to be low/moderate/high (Kappa = 0.93). Inter-rater reliability was also demonstrated across coding completed by both researchers. Utilizing a random number generator, ten of the coded videos were selected for analysis across each variable. The researchers reviewed coding responses to clarify any discrepancies in the coding instrument. Inter-rater agreement was found to be low/moderate/high (Kappa = 0.94).

### 2.4. Statistical Analysis

Composite statistical analyses entailed computing descriptive statistics for the different variables in this study. All observational data collected that delineated the features and characteristics of the videos were synthesized through computing frequencies and percentages of source, format, number of views, length, and content of each video. Across each content category, the number of videos that covered the content was first identified. Next, the total number of views from those videos covering the focused content area was determined. The proportion of total cumulative views was determined by dividing the number of views garnered by the particular videos covering each content area by the total cumulative views generated by all videos (*n* = 4,943,680 views). This process of analysis was conducted for all content categories in the codebook. All analysis was conducted using Excel and Statistical Package for the Social Sciences (SPSS).

## 3. Results

The total number of views for the sample of the 100 most widely viewed videos was 4,943,680. The view counts ranged from 2238 to 891,769. These widely viewed videos were posted between 2009 to 2023. Length of videos ranged from 1.32 min to 89.9 min. The median length of the widely viewed videos was 10.71 min. The interquartile range for the sample ranged from 4.38 min to 27.25 min.

The majority of the videos were posted by consumers and other sources, cumulatively generating greater than 4 million views and ultimately accounting for nearly 85% of the cumulative views. Notably, none of the widely viewed videos were posted by governmental organizations. A total of 29 videos were posted by nongovernmental organization sources that yielded greater than 600,000 views and comprised ~12% of the cumulative views.

A total of 45 of the widely viewed videos were v-blogs, garnering almost 4 million views and nearly 80% of the cumulative views. Although more videos (*n* = 52) were talks by professionals, these videos yielded fewer views (~2 million), representing almost 40% of the cumulative views. There were 30 videos that consisted of testimonials/stories, which generated nearly 1 million views, accounting for approximately 25% of the cumulative views. In addition, there were substantially fewer videos (*n* = 9) that consisted of animations, which also garnered about 1 million views and nearly 25% of the cumulative views. A total of 19 of the widely viewed videos were interviews that yielded ~800,000 views, accounting for almost 17% of the cumulative views. Lastly, documentaries, demonstrations/experiments, tv talk shows/discussion panels, news reports as well as multiple and other formats garnered ~1 million views, which represented less than 20% of the cumulative views. Still images and advertisements were not formats covered in the widely viewed videos.

Anti-depressants, anti-anxiety, antipsychotic and mood stabilizer medications cumulatively received substantial coverage among the widely viewed videos. Nearly 50% of the videos (*n* = 49) presented coverage on antidepressant medications, which garnered greater than 2 million views, accounting for nearly 55% of the cumulative views. Although 13 of the videos covered content on anti-anxiety medications, these videos ultimately generated more than 1 million views, comprising almost 35% of the cumulative views. In contrast, substantially more of the widely viewed videos included coverage of antipsychotic medications (*n* = 41), also comprising greater than 1 million views (about 30% of the cumulative views). There were 18 videos that included content on mood stabilizers, which yielded 1 million views and represented almost 25% of the cumulative views. Stimulants were accounted for in five of the widely viewed videos, generating about 300,000 views, which represented nearly 6% of the cumulative views.

Half of the widely viewed videos presented content on depression, generating more than 3 million views (approximately 70% of the cumulative views). The 37 widely viewed videos that covered anxiety culminated in greater than 1 million views, almost 30% of the cumulative views. Bipolar disorder was covered in 30 of the widely viewed videos, generating almost 2 million views (nearly 40% of the cumulative views). There were 29 videos that delineated content on schizophrenia, which ultimately yielded nearly 1 million views (approximately one-quarter of the total views). Four of the widely viewed videos presented content on schizoaffective disorder, garnering almost a million views (about 20% of the total views). Although there were more videos that included content on ADHD (*n* = 13), these videos culminated in less than a million views (almost 20% of the total views). Eating disorders and personality disorders cumulatively garnered ~1 million views, representing about 20% of the total views. Borderline, other psychotic disorders, and schizoid collectively generated greater than 1 million views (25% of the cumulative views). Six of the widely viewed videos included content on PTSD, yielding about 300,00 views (about 6% of the cumulative views). Lastly, although substance use disorders were covered in marginally more of the widely viewed videos (*n* = 6), these videos garnered less than 200,000 views (nearly 3% of the cumulative views).

There was a wide dispersion in content coverage of side effects from psychotropic medication adherence. Notably, 12 videos covered lack of effect as a side effect, garnering greater than 2 million views (46% of the cumulative views). There were 21 videos that covered weight gain, accounting for more than 1 million views (nearly 35% of the cumulative views). A total of 19 videos presented content on sexual side effects, yielding greater than 1 million views (nearly 30% of the cumulative views). In addition, among the 26 videos that delineated content on fatigue/lack of energy as a side effect, these videos garnered more than 1 million views, comprising nearly 35% of the total views. Insomnia and restlessness collectively generated greater than 3 million views (more than 60% of the cumulative views).

There were 18 videos that included content on individual therapy, garnering more than 1 million views (nearly 25% of the cumulative views). Ten of the videos presented coverage of inpatient psychiatric hospitalizations, which yielded greater than 600,000 views and comprised almost 13% of the cumulative views. Seven of the videos delineated information on cognitive behavioral therapy, generating more than 300,000 views (almost 7% of the cumulative views). Family therapy and informal support group were covered in fewer than 200,000 views, accounting for about 2% of the cumulative views. Group therapy, psychiatric rehabilitation programs, day hospital/partial hospitalization programs, and intensive outpatient programs were not covered in any of the widely viewed videos. Although there were only two videos that presented information on meditation, these videos culminated in almost 700,00 views, comprising 14% of the cumulative views. Content pertaining to seeking support from counselors/therapists, social workers, and psychologists generated ~500,000 views, comprising nearly 10% of the cumulative views. Peer recovery, nature-related resources, community groups, seeking support from primary and subspecialty care providers and psychiatric nurse garnered ~400,000 views, representing about 8% of the cumulative views.

Eight of the widely viewed videos covered content on stigma related to employment, garnering ~1 million views (about 20% of the cumulative views). Although increasingly more of the widely viewed videos covered stigma pertaining to medication dependence, these videos similarly yielded nearly 1 million views, comprising approximately 20% of the cumulative views. There were seven videos that delineated content on stigma contributing to social isolation, generating greater than 800,000 views (approximately 17% of the cumulative views). Three of the widely viewed videos further identified stigma extending to peer network, culminating in greater than 600,000 views (almost 14% of the cumulative views). Distrust in diagnosis and label for life were stigma-related considerations that garnered ~600,000 views, comprising nearly 7% of the total views. Surprisingly, none of the widely viewed videos covered content on stigma perpetuated by society.

Fourteen of the widely viewed videos presented content on medical conditions and illness as factors affecting psychotropic medication adherence, garnering greater than 2 million views (nearly 50% of the cumulative views). Academic stressors were covered in five of the widely viewed videos, generating almost 1 million views (about 20% of the cumulative views). More widely viewed videos (*n* = 10) delivered content on trauma/violence as stressors and triggers impacting psychotropic medication adherence, yielding more than 700 views and approximately 15% of the cumulative views. Two of the widely viewed videos delineated content on workplace-related stressors, accounting for more than 600 views (nearly 13% of the cumulative views). Although familial stressors were included in a marginally higher number of videos (*n* = 5), these videos culminated in greater than 500 views, accounting for almost 11% of the total views. Grief/loss and financial stressors/triggers both cumulatively generated ~200,000 views, comprising almost 4% of the cumulative views. Bullying, child care and life changes were not covered in any of the widely viewed videos. A total of 17 of the widely viewed videos delineated content on individual factors pertaining to quality of life and functioning, garnering about 500,000 views (about 11% of the cumulative views).

Of note, there was not much coverage of access to care considerations for psychotropic medications in the widely viewed videos. Although four of the videos presented content on insurance coverage, cumulatively, these videos generated greater than 300,000 views and accounted for approximately 7% of the cumulative views. Underinsurance considerations were covered in greater than 200,000 videos and represented about 5% of the total views. Access to psychotropic medications for uninsured patients as well as community and street pharmacies for access were covered in less than 2% of the cumulative views.

Although only 10 videos presented content on perceived concerns for taking psychotropic medications, these videos garnered greater than 2 million views and accounted for nearly 50% of the cumulative views. Perceived benefits of medications, severity of mental disorder, and self-efficacy garnered approximately 1 million views collectively, representing about 20% of the cumulative views.

Among only four videos that covered content on pregnancy, these videos garnered ~900,000 views and represented almost 19% of the cumulative views. Surprisingly, concurrent substance recovery, breastfeeding, developmental disabilities, and co-morbid conditions were not accounted for in any of the widely viewed videos. In addition, five videos covered content on health literacy and developmental considerations, which yielded less than 1 million views and represented less than 20% of the cumulative views.

Although only two of the widely viewed videos included content pertaining to adolescents, these videos accounted for more than 200,000 views and comprised nearly 5% of the cumulative views. School-age, middle age and elderly subpopulations collectively garnered less than 35,000 views, accounting for less than 1% of the cumulative views. Content on support networks yielded fewer than 300 views and accounted for less than 6% of the cumulative views. Notably, none of the prevalent subjective and objective measures utilized to assess for medication adherence were covered in the widely viewed videos. Table 1, Table 2, Table 3, Table 4 and Table 5 present a breakdown of number of views and cumulative views for sources, formats and content among the widely viewed videos on psychotropic medication adherence.

## 4. Discussion

A majority of the videos were posted by consumers or healthcare providers with their own YouTube channels. These videos attracted a substantial number of views. Notably, the majority of the videos covered content pertaining to adverse side effects of psychotropic medication use; a scant number of videos covered the benefits of these medications for stabilization. In addition, there was not much coverage on access to care considerations for psychiatric care that extends to medication access. Taking these findings into consideration, it is possible that there is an unbalanced representation of content pertaining to psychiatric treatment in medication management, which certainly has clinical care implications. For patients who are uninsured and underinsured, medication access is not always possible. However, in the meantime, they may seek help from free or low-cost sources. Social media is one of these sources that they can utilize as long as they have access to technology. In addition, social media can represent an open healthcare marketplace with a diverse range of options for care. However, the drawback of social media in our digital era is the reality that the majority of posts are not published by credible organizations (e.g., government, non-profit and for-profit organizations). This reality certainly increases the risk of misinformation and disinformation in our digital era. Nevertheless, it is reassuring that several of the videos were published by independent healthcare providers who seek to be positive influencers on social media to demystify longstanding beliefs about psychiatric treatment.

Notably, none of the prevalent measures to assess for medication adherence were referenced in any of the videos. This could be attributed to the fact that only a small number of videos presented content on research studies, and even among these studies, adherence was not assessed via subjective and objective measures. In many of these videos, the focus was primarily on either demystifying side effects and stigma of psychotropic medications or testimonials/stories shared by consumers or news coverage on the perils of side effects from these medications. It is possible that with more findings from published studies in this domain covered on social media, information will be more globally accessible as a learning tool for patients with psychiatric morbidities and their support networks.

Coverage of a wide dispersion of side effects among the widely viewed videos reinforces the significance of accounting for the unique and diverse experiences of patients prescribed psychotropic medications. As these medications impact individuals differently, ensuring that the coverage of the totality of side effects is critical to assure relevance, support, and precision of information in navigating any of these side effects. There was a significant focus on side effects across the vast majority of the videos, which suggests that the constellation of them on social media could be a target for further intervention to address psychotropic medication adherence in the virtual space.

Given the heavy coverage of side effects from psychotropic medication usage, it is crucial for the medical and public health community to take a more active role on social media in helping to present a more balanced representation of benefits and drawbacks with psychotropic medication usage. In addition, experts in psychiatry can further expand their visibility on social media to disseminate credible knowledge to the general lay population of viewers that these medications impact each patient differently based on a range of genetic, physiological, psychological and environmental factors. It follows that content creators can also engage with these experts and the government in disseminating credible, up-to-date information on psychotropic medication effects in a more engaging way to viewers, based on which kinds of formats across videos yielded the greatest number of views (e.g., v-blogs, talks by professionals, animation, and testimonials). Presenting this practical credible information will also provide more education to patients and their families, which could heighten their knowledge and awareness of these medications and could be an adjunct resource in combination with psychiatric care and treatment through the health system. Platform managers could also integrate this content onto their ten featured videos posted by health sources. Policymakers could also account for access to care and coverage considerations of psychiatric medications delineated across widely viewed content on social media to increase accessibility, affordability, quality and delivery of psychiatric care for both our national and global populations.

In addition, considerations for specialized patient populations were not a central focus of the vast majority of the widely viewed videos. For example, only pregnancy and varying degrees of development were each covered in almost 20% of the videos; however, the total views for each of these unique considerations in navigating psychotropic medication management were still below 1 million. Furthermore, in this sample of the widely viewed videos, all of them were below 1 million views, which contrasts with different studies that have examined coverage of health-related considerations and conditions on YouTube with higher total view counts [27,28,29]. Findings from this study suggest that determinants of psychotropic medication adherence as a health behavior receives under-coverage on social media and warrants further exploration to heighten its coverage as social media continues to trend as a health communication medium in this digital era.

A small number of widely viewed videos covered perceived concerns of taking psychotropic medications; however, the cumulative views for these videos was nearly 50%, which suggests that a selected number of videos yielded the highest views. This finding suggests that there are elements/features of these videos that attracted more views, potentially attributed to their appeal, valence, and relevance to the viewers. It is also possible that since several of these videos were posted by consumers or others who operated their own YouTube channels, there were likely elements about these videos that increased the relatability and acceptability of the content for these viewers. Furthermore, content shared on this selected number of videos among the widely viewed videos could be more in line with the preferences, wishes, goals, values, and needs of individuals prescribed psychotropic medications and their support networks.

From a review of the literature, to date, there are no published studies on psychotropic medication adherence coverage on social media. Many published studies have explored several contextual factors surrounding the complexities of this issue [18,30,31,32,33]. Another future direction for further research is to assess content across different social media platforms to inform intervention in addressing a range of considerations surrounding psychotropic medications as a predictor of treatment adherence for patients with psychiatric disorders.

Notably, antidepressants received substantially more coverage among the widely viewed videos than anti-anxiety, antipsychotic, mood stabilizer and stimulant medications. As depression continues to be one of the leading psychiatric morbidities affecting the world, it is not entirely surprising that depression was covered in almost half of the widely viewed videos [1]. However, anxiety disorders, ADHD, and psychosis, among others, have increased in prevalence over the years, especially amidst the global mental health crisis, which was exacerbated during the global pandemic. It is crucial for future digital health communication on popular social media platforms to equitably cover psychotropic medication categories to reach more viewers with diverse psychiatric conditions, along with their support networks. In addition, given the uptake of digital health, there are implications for increased misinformation, not only pertaining to the COVID-19 pandemic, but also across a range of crises that have emerged and have been exacerbated during the pandemic. Our current global medical health crisis is certainly one of them. Digital health misinformation continues to trend, given the widely permissive and accessible content posted and reviewed by the lay population worldwide [34]. Taking a more critical constructive lens in examining content to ultimately determine whether it is value laden or driven by science is one potential strategy for demystifying misinformation. Integrating critical appraisal and evaluation of social media platforms as education mediums could also form another strategy to identify skewness and distortion in content, thereby contributing towards strengthening a more balanced representation of content from a diversity of perspectives.

There were several strengths and limitations of the present study. Inter-rater and intra-rater reliability were both high, which further increased the precision of coding sources, format, and content among the widely viewed videos. A sample size of 100 videos was sufficient in presenting a prime opportunity to identify trends, patterns, and prevalence in content among the widely viewed videos. One limitation pertained to the study design, which was cross-sectional in nature. The sample of videos were extracted at one point in time. With the nature of social media continuing to evolve with increasingly more published content, drawing a sample of widely viewed videos at a later point in time will likely yield different results from this study, which further delimits replicability in the study design. Future work could also critically examine whether changes in content covered on psychotropic medication adherence align with trends in this domain on both national and global levels. Another limitation of this study was the fact that only videos in English were reviewed by the authors, which could preclude relevant content published in another language.

## 5. Conclusions

With advances in science, technology and medicine alongside the increased prevalence of social media as a health communication medium, it is crucial to assure that content posted on social media for health-related considerations inclusive of different health behaviors is precise, up-to-date and engaging to draw in viewers as the basis to support them with navigating psychotropic medication management. Furthermore, in the meantime, until uninsured and underinsured patients have access to psychiatric care for prescribed medications, assuring that they are aware of options is ever more critical. Fortunately, social media as an educational tool has global reach, and in turn, these patients can uncover more information about the range of options as the basis to decide on the most cost-effective ones as well as the ones that align the most with their preferences, goals, values, needs and concerns. Notably, several videos covered perceived concerns linked to side effects, which could certainly be a target for future intervention across both traditional and non-traditional spaces in primary research.

Digital heath trends across all generations form a prominent audience for the dissemination of digital health content in our contemporary times. Generation Z in particular has grown up during the advancing digital era and were likely one of the subgroups of the lay population greatly exposed to social media content during the pandemic. This generation was also closely affected by the sequalae of the pandemic with respect to the increased emergence and exacerbation of different public health crises during the pandemic, including our global mental health crisis. In addition, it is possible that larger pharmaceutical companies could be predatory, sharing content that is value-laden, skewed and misaligned from promoting optimal psychiatric care. It follows that increased visibility of experts in psychiatry and the behavioral and social sciences on social media could mitigate the uptake of misinformation surrounding psychiatric medication adherence.

Successive content analyses could also yield more information over time on any changing trends in coverage of content on psychotropic medication adherence and subsequent engagement among viewers on social media. Future work could further examine topics covered and uncovered to ultimately determine resource allocation and outreach efforts in supporting any of these topics as further points of education and communication to the lay population. Coverage on social media of considerations surrounding psychiatric care yields promise in heightening parity in the consideration of psychiatric care as equivalent to medical care. It follows that integrating up-to-date, credible and accurate content could help achieve this parity. Deliverers of content are pivotal in this knowledge dissemination. Pairing them with content creators and platform managers could strengthen the production and precision of content along with the engagement and acceptability of content among viewers, thereby promoting a downstream effect that has the potential to reach individuals with mental illness and their networks worldwide.

## Figures and Tables

**Table 1:** Frequencies, View Counts, and Cumulative View Count Percent of Widely Viewed Psychotropic Medication Adherence Videos by Upload Source.

Classification of the Source of Video Upload	*n*	View Count	Cumulative View Count Percent (%)
Other	34	2,346,759	47.47
Consumer	22	1,819,187	36.80
Organizational	29	617,939	12.50
Governmental	0	0	0

**Table 2:** Frequencies, View Counts, and Cumulative View Count Percent of Widely Viewed Psychotropic Medication Adherence by Format.

Format	*n*	View Count	Cumulative View Count Percent (%)
V-Blog	45	3,843,301	77.74
Talk By Professional	52	1,935,802	39.16
Animation	9	1,292,235	26.14
Testimonial/Story	30	1,230,845	24.90
Interview	19	826,979	16.73
Other Formats	6	421,335	8.52
TV Talk Show/Discussion Panel	5	379,862	7.68
Documentary	2	75,337	1.52
Multiple Formats	4	144,451	2.92
News Report With Anchor	2	43,874	0.89
Demonstration/Experiment	1	3986	0.081
Still Images	0	0	0
Advertisement	0	0	0

**Table 3:** Frequencies, View Counts, and Cumulative View Count Percent of Widely Viewed Psychotropic Medication Adherence by Psychiatric Diagnosis.

Psychiatric Diagnosis	*n*	View Count	Cumulative View Count Percent (%)
Depression	50	3,657,150	73.98
Bipolar	30	1,903,647	38.51
Anxiety	37	1,376,709	27.85
Schizophrenia	29	1,333,308	26.97
Schizoaffective Disorder	4	959,274	19.40
ADHD	13	937,171	18.96
Eating Disorder	5	674,712	13.65
Other Psychotic Disorder	13	634,202	12.83
Personality Disorder	2	366,296	7.41
Schizoid	1	354,335	7.17
PTSD	6	313,985	6.35
Borderline	3	296,768	6.00
Substance Use Disorder	7	159,997	3.24
Disruptive Mood Dysregulation Disorder	0	0	0

**Table 4:** Frequencies, View Counts, and Cumulative View Count Percent of Widely Viewed Psychotropic Medication Adherence by Typology of Psychotropic Medication.

Psychotropic Medication Category	*n*	View Count	Cumulative View Count Percent (%)
Antidepressant	49	2,713,110	54.88
Anti-anxiety	13	1,730,560	35.00
Antipsychotic	41	1,551,987	31.39
Mood Stabilizer	18	1,162,126	23.51
Stimulant	5	311,768	6.30

**Table 5:** Frequencies, View Counts, and Cumulative View Count Percent of Widely Viewed Psychotropic Medication Adherence by Side Effects.

Side Effects	*n*	View Count	Cumulative View Count Percent (%)
Lack of Effect	12	2,273,385	45.99
Fatigue/Lack of Energy	26	1,727,257	34.94
Restlessness	13	1,716,776	34.73
Weight Gain	21	1,677,349	33.93
Insomnia	16	1,643,755	33.25
Sexual Side Effects	19	1,619,310	32.76
Dryness	17	1,537,804	31.11
Tremors	13	1,532,536	31.00
Headache	16	1,459,421	29.52
Gastrointestinal Distress	15	1,443,185	29.19
Seizures	11	1,377,610	27.87
Dizziness	12	1,362,297	27.56
Hypotension	7	1,312,237	26.54
Fever	6	1,292,659	26.15
Hypertension	5	1,272,328	25.74
Tachycardia	4	1,267,907	25.65
Self-Harm	3	1,146,209	23.19
Weight Loss	9	1,143,003	23.12
Sleep Issues	19	1,140,208	23.06
Blurred Vision	11	1,130,917	22.88
Sweat/Perspiration	10	1,068,253	21.61
Sudden Changes of Mood	4	1,042,816	21.09
Urinary Retention	11	1,008,142	20.39
Constipation	9	1,006,094	20.35
Heart Attack	4	948,772	19.19
Hopelessness	2	937,723	18.97
Dehydration	6	928,116	18.77
Low Platelets	2	901,654	18.24
Despair	1	891,769	18.04
Loss of Appetite	7	807,774	16.34
Hallucinations	9	708,187	14.33
Tardive Dyskinesia	10	705,072	14.26
Cognitive Impairment	18	617,895	12.5
Nausea	14	535,410	10.83
Irritability	5	492,290	9.96
Diminished Intellect	4	479,568	9.70
Emotional Blunting	9	470,644	9.52
Myocarditis	3	387,273	7.83
Ataxia	3	373,078	7.55
Tingling	1	354,335	7.17
Chills	1	354,335	7.17
Delusions	4	256,141	5.18
Rash	9	233,064	4.71
Impulsivity	2	197,599	4
Memory Loss	5	190,437	3.85
Heart Palpitations	5	182,845	3.7
Photosensitivity	2	146,907	2.97
Kidney Function	5	96,517	1.95

## Data Availability

Not applicable.
